# Clinical and Genetic Characteristics of Pediatric Patients with Inflammatory Bowel Disease Transitioning to Adult Medicine: A Single-Center Ten-Year Experience

**DOI:** 10.3390/jcm14113741

**Published:** 2025-05-27

**Authors:** Giammarco Mocci, Giorgia Orrù, Francesca Maria Onidi, Mara Corpino, Antonella Marongiu, Giovanni Maria Argiolas, Matteo Runfola, Romina Manunza, Giorgia Locci, Elisabetta Tamponi, Teresa Zolfino, Paolo Usai Satta, Alessandro Muscas, Rossano Rossino, Salvatore Savasta, Mauro Congia

**Affiliations:** 1 Division of Gastroenterology, “Brotzu” Hospital, 09047 Cagliari, Italy; francescamariaonidi@aob.it (F.M.O.); teresa.zolfino@aob.it (T.Z.); paolousai@aob.it (P.U.S.); 2 Department of Medical Sciences and Public Health, University of Cagliari, 09042 Cagliari, Italy; giorgiaorru1996@tiscali.it; 3 Pediatric Clinic and Rare Disease, Microcitemic Asl8, 09121 Cagliari, Italy; mara.corpino@aob.it (M.C.); salvatore.savasta@unica.it (S.S.); maurocongia1957@gmail.com (M.C.); 4 Neonatology SS Trinità Hospital, 09121 Cagliari, Italy; giammarco.mocci@gmail.com; 5 Division of Radiology, “Brotzu” Hospital, 09047 Cagliari, Italy; gio.argiolas@gmail.com; 6 Division of Surgery, “Brotzu” Hospital, 09047 Cagliari, Italy; matteo.runfola@gmail.com (M.R.); romina.manunza@aob.it (R.M.); 7 Unit Pathologic Anatomy, “Brotzu” Hospital, 09047 Cagliari, Italy; giorgia.locci@aob.it (G.L.); elisabetta.tamponi@aob.it (E.T.); 8 Division of Pediatric, “Brotzu” Hospital, 09047 Cagliari, Italy; alessandro.muscas@aob.it; 9 Department of Medical Science and Public Health, Cagliari University, Monserrato, 09042 Cagliari, Italy; moccigiammarco@gmail.com

**Keywords:** inflammatory bowel disease, adolescents, young adults, transition care, transition to adult care inflammatory bowel disease-basic, epidemiology, natural history, treatment

## Abstract

**Background/Objectives**: Inflammatory bowel diseases (IBDs) comprise a group of chronic idiopathic disorders, including ulcerative colitis (UC), Crohn’s disease (CD), and indeterminate colitis (IC). Complex genetic factors, in addition to environmental triggers, have been shown to play a fundamental role in the pathogenesis of IBD, contributing to disease susceptibility. The transition of adolescents with inflammatory bowel disease (IBD) to adult care represents a significant challenge for patients, their families, and healthcare providers. Approximately 25% of individuals with IBD receive a diagnosis before the age of 16, and this population is at increased risk for adverse clinical outcomes. As a result, the transition of care has garnered substantial attention in the scientific and clinical communities over the past decade. This study aims to analyze a cohort of pediatric Sardinian patients with IBD to assess clinical characteristics at diagnosis and at the time of transition and determine potential correlations between NOD2/CARD15 gene variants and HLA class II with the disease phenotype. **Methods**: From January 2014 to August 2024, we performed an observational, cross-sectional study that included pediatric patients with IBD enrolled in the only pediatric IBD reference center in Sardinia. Data were obtained from the patients’ medical records and from a questionnaire administered at the inclusion visit. In addition, we genotyped a portion of our cohort for the Leu1007fsinsC (SNP13), Gly908Arg (SNP12), and Arg702Trp (SNP8) variants of the NOD2/CARD15 gene, as well as for HLA-DRB1, -DQA1, and -DQB1 class II genes. The obtained results were compared with pediatric data from the national epidemiological IBD registry and existing literature. **Results**: Seventy-one IBD patients were enrolled (UC 43, CD 28, M 34, F 37). Median age at diagnosis was 12.2 years (IQR 2–17). After a median disease duration of 5 years (IQR: 1–16), only three UC patients experienced proximal extension of proctitis or left-sided colitis, and no CD patients experienced new localizations of disease. Fifteen patients developed extraintestinal manifestations. No significant difference was found in median diagnostic delay (DD) between UC [4 months (IQR: 1–84)] and CD patients [4.5 months (IQR: 1–48)]. At the transition visit, overall, twenty-nine patients (42%) were exposed to one biologic agent (vs. 3% at baseline; *p* < 0.02); 3 patients (4%) were exposed to two or more biologic agents. 7% of patients (5/71) underwent surgery. By comparing the distribution of NOD2/CARD15 SNPs between pediatric patients and an adult CD population, we found a significant association between gene allelic variants and pediatric onset (*p* = 0.00048). Our study also revealed a statistically significant association between Sardinian pediatric patients carrying NOD2/CARD15 mutations and early-onset CD (*p* < 0.009492), along with a stenosing phenotype (*p* < 0.024) and increased surgical risk (*p* < 0.026). No significant associations were observed between HLA class II alleles and IBD in our population. **Conclusions**: Our results provide important insights into the clinical and epidemiological features of the pediatric IBD population. In addition, our study highlights the significant role of NOD2/CARD15 gene polymorphisms in pediatric onset CD. These variants influence the age of onset and disease phenotype, characterized by greater severity and a higher risk of surgical intervention in pediatric patients.

## 1. Introduction

Inflammatory bowel diseases (IBD) encompass chronic idiopathic conditions, including ulcerative colitis (UC), Crohn’s disease (CD), and indeterminate colitis (IC). IBD exhibits a bimodal distribution with onset typically between 10 and 20 years and a second peak between 50 and 80 years. Approximately 25% of IBD cases are diagnosed during childhood [1,2].

In the pediatric setting, the majority of available data come from studies conducted in North America and Northern Europe, while data from Southern Europe remain limited. In Europe, the annual incidence of pediatric UC ranges from 1 to 4 cases per 100,000 individuals and remains stable, whereas the incidence of pediatric CD is 9–10 cases per 100,000 individuals [3]. In UC, the male-to-female ratio ranges from 0.51 to 1.58, suggesting no gender specificity, whereas in CD there is a slight male predominance (F/M ratio: 0.82) [4].

A recent study by SIGENP (Italian Society of Gastroenterology, Hepatology and Pediatric Nutrition) on the Italian epidemiological trends of pediatric IBD from 2009 to 2018 reported an increased incidence in Italy, with a minimal rate of 1.59–2.04 per 100,000 inhabitants under 18 years [5].

Relevant differences between childhood-onset and adulthood-onset IBD have been observed, with children showing more extensive anatomic involvement for both UC and CD. In adolescents with ulcerative UC, the disease is more likely to involve the entire colon (pancolitis), occurring in approximately 67% of cases. In contrast, adults with UC more commonly have disease limited to the left side of the colon, with pancolitis present in only about 39% of cases. CD, which can affect any part of the gastrointestinal tract, shows a different pattern in children. Pediatric CD more frequently involves the ileum and colon together or the small intestine alone—accounting for roughly 40% of cases. Isolated colonic involvement is less common in children, occurring in only about 10% of cases.

Pediatric CD also differs from the adult also for a more severe phenotype, including extensive and aggressive intestinal involvement, a higher prevalence of penetrating or stricturing complications, and a greater risk of perianal disease. Moreover, it is frequently associated with significant growth impairment and delayed puberty, which reflect the systemic inflammatory burden and the impact on nutritional status and hormonal development [6,7].

Given that approximately 25% of IBD cases are diagnosed before the age of 16—and that these individuals are at an increased risk for unfavorable clinical outcomes—there has been a growing focus on transition care over the past decade. The period of transition is crucial to the well-being of adolescents with chronic conditions and their caregivers. Several studies have highlighted that the transition process can sometimes lead to reduced adherence to therapy and fewer medical visits, resulting in increased disease flare-ups [8]. The process aims at the development of self-management, self-efficacy, and self-advocacy. Meeting these expectations requires the development of additional competencies, including effective communication, decision-making, and assertiveness [9,10,11].

The term “transition” refers to the process of transferring the patient and their entire clinical history from the pediatric gastroenterologist to the adult gastroenterologist [12]. In recent years, guidelines from different gastroenterological societies have emerged for the transition process from pediatric to adulthood [13]. Currently, there is no uniform model for successful transition, and the transition process follows different approaches across countries. For instance, in Canada and Europe, the mandatory transfer usually takes place by the age of 18, while in the United States, young adults can remain on their parents’ insurance until age 26, which may delay the development of independence [14]. However, it is important to emphasize that the transition process does not only depend on the patient’s age but also on psychological developmental maturity and therefore requires preparation and education starting from early adolescence [15,16]. Likewise, the European Crohn’s and Colitis Organization Topical Review on Transitional Care in Inflammatory Bowel Disease recommends choosing a transition model based on the local availability of resources, such as the option of single or multiple joint appointments, the site of meetings, and the number of participants involved [13].

Finally, the accurate diagnosis of IBD and differentiation between UC and CD rely on a combination of clinical, laboratory, endoscopic, histological, and radiological data. Concerning the genotype-disease phenotype association, some studies have shown an increased prevalence of stricturing disease [17] and a higher risk of undergoing surgery in children carrying at least one NOD2 variant [18], while other studies have found no association [17].

There is a well-established association between HLA and IBD, supported by GWAS studies that have mapped the HLA system within the IBD3 region. Family studies suggest that HLA may contribute to 10–33% of the genetic risk for Crohn’s disease and 64–100% for ulcerative colitis. Therefore, the genetic significance of HLA is more pronounced for UC than for CD, in contrast to NOD2/CARD15 [19].

This study seeks to examine a group of pediatric IBD patients from Sardinia in order to evaluate their clinical features at diagnosis and at the time of transition. Moreover, we aimed to investigate potential associations between NOD2/CARD15 gene variants and HLA class II alleles in relation to the disease phenotype.

## 2. Materials and Methods

### 2.1. Study Design and Population

We performed a single-center, observational, cross-sectional study including pediatric patients with IBD followed at the Gastroenterology unit of the Pediatric Clinic for Rare Diseases of the Microcitemico A. Cao Hospital in Cagliari, Sardinia, and transitioned to the adult center between January 2014 and August 2024. In most cases, the transition was organized through one or more joint meetings. Sardinia is an island in the Mediterranean Sea with a population of approximately 1.6 million. Its regional healthcare system is part of the Italian National Health System (INHS), which guarantees universal health coverage for all residents, including free access to general practitioner services and hospital care. The INHS also ensures free access to medications and specialist care for individuals with chronic diseases, provided that the diagnosis is certified by a specialist and recorded in the Disease-Specific Payment Exemptions Register (DPER).

Patient data were anonymized and recorded in a centralized database for analysis. All participants provided written informed consent. The study was approved by the Ethics Committee (Protocol No. PG/2020/10905) and conducted in compliance with the Declaration of Helsinki.

### 2.2. Inclusion and Exclusion Criteria

We included pediatric patients (<18 years old) with an established diagnosis of IBD, based on standard clinical, endoscopic, and histologic criteria [20,21]. Diagnosis was made at least 3 months before the study inclusion, and the minimum follow-up time was 1 month. Exclusion criteria was inability to provide consent.

Demographic and clinical data at the time of diagnosis and at the point of transition were collected using a standardized, shared database. The variables included sex, date of birth, lifestyle factors (such as smoking and alcohol consumption), personal and/or family history of neoplasia, vaccination status (hepatitis B virus [HBV], human papillomavirus [HPV], and *Streptococcus pneumoniae*), year and age at diagnosis, disease extent and phenotype at both diagnosis and transition, presence of extraintestinal manifestations (EIMs), treatment history with IBD-related medications (mesalazine [5-ASA], corticosteroids, immunosuppressants, and biologic agents), and history of surgery.

Disease extension and regression were defined as a proximal progression or distal regression from the initial extent at diagnosis, respectively, as determined by endoscopy.

#### 2.2.1. Clinical Assessment

At diagnosis, disease severity was determined using clinical scores, the Pediatric Ulcerative Colitis Activity Index (PUCAI) for UC [17], and the Pediatric Crohn Disease Activity Index (PCDAI) for CD [18]. Disease localization and phenotype were described using the Paris Classification [22,23,24].

To analyze the age at diagnosis, patients were classified as Very Early Onset (VEO) if the disease onset occurred between 2 and 5 years, Early Onset (EO) if between 6 and 9 years, and Pediatric Onset (PO) if between 10 and 17 years.

At transition, the extent and phenotype of disease were determined using the Montreal classification. Severity was determined using the Mayo score in UC patients and the HBI in CD patients [25,26,27].

#### 2.2.2. Genetic Analysis

During routine follow-up visits, blood samples were collected from 45 out of 71 pediatric patients after obtaining informed consent, with the aim of detecting NOD2/CARD15 mutations and performing HLA class II typing.

Genomic DNA was extracted from peripheral blood samples collected in EDTA tubes using the automated BioRobot EZ1 DSP system (QIAGEN GmbH, Hilden, Germany).

The three major single nucleotide polymorphisms (SNPs) of the NOD2/CARD15 gene—SNP8 (Arg702Trp), SNP12 (Gly908Arg), and SNP13 (Leu1007fsinsC)—were analyzed by polymerase chain reaction (PCR) followed by restriction enzyme digestion and separation on a 2% agarose gel stained with ethidium bromide, with visualization under UV light [28].

To compare the frequency of NOD2/CARD15 mutations between pediatric and adult patients with Crohn’s disease, DNA samples from 110 adult CD patients, provided by Prof. Paolo Usai (University of Cagliari), were included in the analysis. HLA-DRB1, -DQA1, and -DQB1 genotyping was performed using sequence-specific primers PCR (SSP-PCR) with low-resolution typing kits (Olerup SSP AB, Stockholm, Sweden). HLA class II haplotypes were inferred based on established patterns of linkage disequilibrium specific to the Sardinian population, as described in previous studies [29,30]. A control group of 627 healthy Sardinian subjects, previously genotyped for HLA class II alleles, was used for comparison [31].

#### 2.2.3. Statistical Analysis

GraphPad Prism 8.4.3 was used to analyze the data. Normally distributed variables were expressed as mean (SD) and tested using the *t*-test. For non-normally distributed variables, expressed as median (IQR), the Mann–Whitney U test was used. Categorical data were compared using the chi-squared test or, when appropriate, Fisher’s exact test. *p*-values less than 0.05 were considered statistically significant.

## 3. Results

### 3.1. Sex and Age at Diagnosis and EIMs

A total of 71 Sardinian patients, comprising 34 boys and 37 girls, were included in this study (Table 1). Among them, 44 were diagnosed with UC (15 were males, 29 were females, male-to-female ratio 1:2), and 27 with CD (19 boys, 8 girls, male-to-female ratio: 2.5:1). The age at diagnosis ranged from 2 to 17 years, with a mean age of 12.2 years.

For UC, 2 children (4.5%) were classified as VEO, 6 (13.6%) as EO, and 36 (81.8%) as PO.

For CD, no patients had onset below 5 years, five (18.5%) had onset between 6 and 9 years, and 22 (81.4%) were classified as PO.

Finally, fifteen patients (21.1%) developed extraintestinal manifestations (EIMs), the most frequent being articular (8/71, 11.2%) and hepatobiliary (8/71, 11.2%, of which 5 patients had primary sclerosing cholangitis), followed by cutaneous (1.41%) and ocular (1.41%).

### 3.2. Disease Extent, Phenotype and Severity

At diagnosis, using the Paris classification [24], the most common localization in UC patients was pancolitis (E4), affecting 26 patients (59%). This was followed by 7% of patients with localization to the rectum, sigmoid, and descending and transverse colon (E3), 25% with localization to the rectum, sigmoid, and descending colon (E2), and 9% with disease localized only in the rectum (E1).

At the transition visit, using Montreal classification, after a median disease duration of 5 years (IQR 1–16), 3 patients (7%) experienced proximal extension of proctitis or left-sided colitis: 2 patients from E1 to E2, 1 from E2 to E3.

In CD, at diagnosis, the most frequent localizations were ileal (L1) and ileocolonic (L3), with 12 patients each (44.4%), followed by colonic (L2) in only 3 patients (11.1%). Additionally, 4 patients (14.8%) of our CD cohort presented with perianal disease localization. At transition, no CD patients experienced new localizations of disease.

With regard to behavior, at diagnosis the most common phenotype in our patients was non-stricturing/non-penetrating (B1, 74.07%), while only 25.93% exhibited a stricturing (B2) phenotype. At transition, no CD patients experienced phenotype changes.

Disease severity at the transition visit was valued using clinical scores (PUCAI for UC and PCDAI for CD). For UC, 25% of patients exhibited mild disease activity, 67% moderate, and 8% severe. In CD, 28% had mild disease activity, 44% moderate, and 28% severe.

### 3.3. Diagnostic Delay

Diagnostic delay was defined as the time interval between symptom onset and the confirmed diagnosis. Data on diagnostic delay were available for 68 patients (41 with ulcerative colitis [UC] and 27 with Crohn’s disease [CD]). No significant difference was found in the median diagnostic delay between UC and CD patients (*p* = 0.8431): 4 months (IQR: 1–84) for UC and 4.5 months (IQR: 1–48) for CD. In addition, 22% (15/68) and 11.7% (8/68) of patients experienced a diagnostic delay of ≥12 and ≥24 months, respectively, with no significant differences between UC and CD (*p* = 0.766 and *p* = 0.463, respectively).

### 3.4. Medical and Surgical Treatment

Details on medical therapy are provided in Table 2. The most common therapy at the inclusion visit was 5-ASA, which was used on 47 patients (66.2%), with a higher, but not statistically significant, percentage in UC compared to CD (72% vs. 55%, *p* = 0.217).

Six patients (8.5%) were being treated with corticosteroids, either systemic or with low bioavailability, whereas only one patient was receiving topical therapy at the transition visit.

Azathioprine was used by twenty-two patients (40.8%), six of whom were on a combination regimen with infliximab (IFX).

At the transition visit, 29 patients (42%) were exposed to at least one biologic agent, compared to only 3% at baseline (*p* < 0.02). Additionally, three patients (4%) were exposed to two or more biologic agents. At study conclusion, infliximab was the most commonly used anti-TNFα biologic (19/71, 26.7%), followed by adalimumab (10/71, 14.1%).

Finally, a total of five patients (7%), all with Crohn’s disease (CD), underwent ileocecal resection.

### 3.5. Genetic Features

The results of genetic analysis are summarized in Table 3.

A total of 45 out of 71 patients (21 with CD and 24 with UC) were genotyped for three main NOD2/CARD15 gene variants. Among the CD patients, nine (42.9%) carried at least one disease-associated mutations: four were heterozygous for Leu1007fsinsC (SNP13), 3 for Gly908Arg (SNP12), and two for Arg702Trp (SNP8). None of the UC patients harbored any of these variants.

We next compared the frequency of NOD2/CARD15 variants in our pediatric CD cohort (n = 21) with that of a previously genotyped Sardinian adult CD population (n = 110). A statistically significant difference was observed (*p* = 0.00048), with only 13 out of 110 (11.8%) adult CD patients carrying NOD2/CARD15 variants, compared to 9 out of 21 (42.9%) in the pediatric cohort.

To evaluate whether the presence of NOD2/CARD15 mutations was associated with an earlier onset of CD, we compared the age at diagnosis between mutated and non-mutated pediatric patients. A significant association was found, with mutation carriers presenting at a younger age (*p* = 0.009).

We further investigated whether NOD2/CARD15 mutations were linked to disease phenotype. A statistically significant association was detected between mutation status and disease severity (*p* = 0.024).

Notably, the need for surgical intervention was also significantly higher among CD children carrying NOD2/CARD15 mutations compared to non-carriers (*p* = 0.026).

Regarding HLA class II genotyping, no significant differences were observed in haplotype or genotype frequency distributions between our cohort of pediatric IBD patients and the healthy Sardinian control population.

## 4. Discussion

In this study, we summarized the main characteristics and natural history of IBD in a pediatric population from Sardinia, Italy. We included data on genetic characteristics, demographic information, disease extension, EIMs, and both medical and surgical treatment. It is important to know the characteristics of our population in order to understand and interpret the results of our study and compare them with data published in the literature.

First, in our cohort of 71 patients, the average age of disease onset was 12.2 years, with UC being more common than CD (61.9% vs. 38.1%). The predominance of UC was especially marked in the Very Early Onset (0–5 years) and Early Onset (6–9 years) categories, while it was less pronounced in Pediatric Onset (10–17 years), with a UC/CD ratio of 0.8 in the latter age range. Additionally, a gender predominance was observed in both diseases: UC was more frequently diagnosed in females, whereas CD showed a higher prevalence among males. These findings are consistent with other Italian pediatric IBD studies [5,32,33], but differ from those reported in European cohorts [3,34]. Notably, the average age at disease onset in our cohort was lower than in other European studies [2,34,35], likely reflecting the referral pattern in which patients approaching adulthood are more often directed to adult care centers.

Second, disease extent in IBD is a critical factor, as it serves as an indicator of disease severity and influences therapeutic decision-making. In particular, patients with UC who present with pancolitis at diagnosis tend to experience a more severe disease course and often require more intensive medical and surgical interventions. In contrast, distal UC is generally associated with a more favorable prognosis [36]. Our study found that the predominant disease localization in UC was pancolitis (71% of patients), which is not surprising since pediatric UC typically presents with more extensive involvement than adult forms, with pancolitis in 60–80% of cases [36]. In CD, the most common localization was ileocolonic (L3), in line with Italian study data [5,6,7,8,9,10,11,12,13,14,15,16,17,18,19,20,21,22,23,24,25,26,27,28,29,30,31,32,33,34,35,36,37]. Furthermore, 17% of our CD patients had perianal disease, which is associated with a more severe disease course. This finding is consistent with literature reporting perianal localization in 8–26% of CD patients [38].

Regarding disease behavior, the Paris Classification revealed that the non-stricturing/non-penetrating phenotype (B1) was the most common among our CD patients, in line with European datasets [39]. The stricturing phenotype (B2), which is associated with a more severe disease and a higher risk of surgery, was present in 24% of our patients. This is similar to the 26% reported in pediatric CD patients in other studies [8,13].

Extraintestinal manifestations (EIMs) are common in IBD and can involve almost every organ system [40]. The true prevalence and burden of EIMs in IBD have not been fully elucidated, with estimates ranging from 15% to 50%. This variability is largely due to the absence of prospective studies and the heterogeneity in definitions of EIMs across studies. Notably, many definitions exclude nonspecific manifestations such as arthralgia, as well as conditions like anemia, hepatitis, pancreatitis, and osteoporosis, which may or may not be directly related to IBD [41]. Compared to adult data, there is a limited amount of research on pediatric populations. However, some studies suggest that younger patients with IBD experience a higher prevalence of EIMs, which are more commonly observed at the time of diagnosis in pediatric patients than in adults [37,42,43]. In our cohort, 21.1% of patients experienced EIMs, a higher percentage than reported in the Italian register [5]. The most common EIMs were articular and hepatobiliary (both 11.2%) manifestations, the first ones more frequent in Crohn’s disease and the second ones, in particular sclerosing cholangitis, in UC patients.

Diagnostic delay (DD) was defined as the time interval between the onset of IBD-related symptoms and the establishment of a definitive diagnosis [44]. DD is generally shorter in pediatric patients compared to adults. For instance, in a Swiss cohort of 1550 patients with Crohn’s disease—25% diagnosed before the age of 18 and the remainder in adulthood—the median DD was 3 months in the pediatric group versus 6 months in the adult group [45]. This difference is largely expected and may be explained by differing health-seeking behaviors: while adult patients may delay medical consultation and resort to self-medication, parents are typically more prompt in seeking medical attention for their children. Furthermore, in pediatric cases, Crohn’s disease may present with growth failure, a red flag that often prompts further diagnostic evaluation [46]. In our cohort, the mean diagnostic delay was 4 months in the overall cohort, 4.5 months in CD patients, and 4 months in the UC cohort. Interestingly, our data were in line with the results of the SIGENP registry [5] and of a recent review that included 26 studies in the final analysis, with a pooled cohort of 7030 children [37]. Moreover, the proportions of patients with a DD ≥ 12 and 24 months were, respectively, 22% (15/68) and 11.7% (8/68), with no significant difference between CD and UC (*p* = 0.7662 and *p* = 0.4630, respectively).

Regarding therapy, 5-ASA remains the primary treatment for UC, with 72% of patients taking it at the transition visit. Interestingly, despite current guidelines [47,48], 5ASA remains widely used in Crohn’s disease [49,50]. In a recent Italian web survey investigating the current use of mesalazine in clinical practice by young gastroenterologists and GI trainees, more than 70% of both IBD and non-dedicated IBD physicians use mesalazine to prevent clinical recurrence in CD [51]. Currently, only two biologic therapies, both anti-TNF agents—infliximab and adalimumab—are approved by the European Medicines Agency (EMA) for the treatment of children with CD, having received approval in 2006 and 2014, respectively. For children with UC, these therapies were approved in 2011 and 2021 [52].

In recent years, there has been an increasing and increasingly early use of these drugs, even in pediatric settings, especially in CD [53,54]. In our cohort, at the transition visit, 42% of patients were exposed to one biologic agent, and 4% were exposed to two or more biologic agents. Infliximab was the most common anti-TNFα biologic used (26.7%), followed by adalimumab (14.1%).

Finally, another important finding seen in this study is that in approximately one-third of patients, IFX was used in combination with an immunomodulatory drug.

We also analyzed the genetic characteristics of our cohort. Sardinians represent an ethnically homogeneous population whose genetic background has been extensively studied by our group and others [55,56,57]. This provides a unique context for investigating genotype–phenotype correlations in IBD. To our knowledge, no systematic studies have addressed the prevalence of NOD2/CARD15 variants in Sardinian children with Crohn’s disease (CD). Since 2007, all pediatric patients diagnosed with CD at our center have undergone routine NOD2/CARD15 genotyping. Here, we present the first comprehensive analysis of this genotyping effort, covering a 10-year period (2014–2024). As expected, the frequency of NOD2/CARD15 variants was higher in pediatric CD patients compared to the general Sardinian population, in agreement with numerous studies across diverse ethnic groups [58]. In line with the literature, no NOD2/CARD15 gene mutations were found in UC patients [59,60].

We compared our Sardinian pediatric population to an adult population with CD, previously genotyped for NOD2/CARD15 SNPs. We found a statistically significant difference (*p* < 0.0005), suggesting that NOD2/CARD15 gene variants play a crucial role in determining early disease onset. This hypothesis was further supported by a statistically significant comparison (*p* < 0.009) between the age at onset in pediatric patients with mutations and those without mutations. The relationship between NOD2/CARD15 gene variants and early CD onset has been reported in various studies [61,62] and may be attributed to the idea that in early-onset diseases, the genetic component plays a more significant role than the environmental component [63].

Finally, we evaluated the impact of genetic mutations on disease phenotype and the risk of surgical intervention. The presence of NOD2/CARD15 variants was significantly associated with a stricturing phenotype, observed in 55% of mutation-positive patients. This phenotype was linked to a more severe clinical course, requiring surgery in five cases. In contrast, only one patient without NOD2/CARD15 mutations (9%) exhibited a stricturing phenotype (*p* < 0.024). Findings in the literature regarding this association vary, having been reported in both adult and pediatric populations [64,65,66,67,68]. This variability may be explained by differences in environmental exposures and genetic backgrounds across the studied cohorts.

Regarding the risk of surgery, we found that patients with NOD2/CARD15 variants were at a higher risk (*p* < 0.026) compared to patients without mutations. This finding is consistent with existing literature, reflecting a more severe disease course and an increased risk of complications associated with these mutations [18,68,69].

Although associations between HLA class II alleles and IBD have been reported in the literature [70,71,72], our study did not replicate these findings. We observed no statistically significant associations between pediatric IBD patients and a Sardinian control population. This discrepancy may reflect differences in allelic frequencies between the Sardinian population and those previously examined in other studies.

This study presents several strengths. It is based on data from the only pediatric gastroenterology center in Sardinia, thus providing a highly representative sample of the regional pediatric IBD population. Furthermore, the use of detailed clinical records—rather than administrative data from national registries—allows for a more nuanced understanding of patient characteristics and management, particularly regarding aspects of care that are most relevant to clinical practice.

Nevertheless, the study has some limitations. Its retrospective design prevents standardized follow-up in terms of both clinical assessments and endoscopic evaluations. Additionally, the relatively small sample size may limit the statistical power to detect certain associations.

## 5. Conclusions

In conclusion, this study represents one of the first investigations conducted in Italy, offering valuable insights into the clinical and epidemiological characteristics of the pediatric IBD population.

Our data are in line with Italian and European data. In addition, our study highlights the significant role of NOD2/CARD15 gene polymorphisms in pediatric onset CD. These variants influence the age of onset and disease phenotype, characterized by greater severity and a higher risk of surgical intervention in pediatric patients.

## Figures and Tables

**Table 1:** Patients features at transition.

	OVERALL n = 71	UC n = 44	CD n = 27
**Gender, male n (%)**	34 (47.88)	15 (34.1)	19 (70.37)
**Age at diagnosis, yr, mean (range)**	12.257 (2–17)	12.03 (2–17)	12.25 (7–17)
VEO n (%)	2 (7.04)	2 (4.5)	0 (0.00)
EO n (%)	11 (15.49)	6 (13.63)	5 (18.52)
PO n (%)	55 (77.46)	36 (81.82)	22 (81.48)
**Disease duration, yr, median (IQR)**	5 (1–16)	5 (1–16)	5 (1–16)
**EIMS**	15 (21.12)	9 (20.45)	6 (22.22)
Articular	8 (11.27)	3 (6.81)	5 (18.51)
Cutaneous	1 (1.41)	0 (0.00)	1 (3.70)
Ocular	1 (1.41)	0 (0.00)	1 (3.70)
Hepatobiliary	8 (11.27)	7 (15.91)	1 (3.70)
Others	1 (1.41)	1 (2.27)	0 (0.00)
**Appendectomy, n (%)**	1 (1.41)	1 (2.27)	0 (0.00)
**Surgery n (%)**	5 (7.04)	0 (0.00)	5 (18.51)
**Smoking n (%)**	4 (5.63)	3 (6.81)	1 (3.70)
**Montreal classification UC**			
Proctitis n (%)		2 (4.55)	
Left sided n (%)		12 (27.27)	
Extensive n (%)		30 (68.18)	
**Montreal classification CD**			
Isolated ileal n (%)			12 (44.44)
Isolated colonic n (%)			3 (11.11)
Ileocolonic n (%)			12 (44.44)
Isolated UGI n (%)			0 (0.00)
Perianal disease n (%)			4 (14.81)
**Behavior**			
Non stricturing/non penetrating n (%)			20 (74.07)
Stricturing n (%)			7 (25.93)
Penetrating n (%)			0 (0.00)

VEO: very early onset; EO: early onset; PO: pediatric onset, EIMS: extraintestinal manifestations.

**Table 2:** Medical treatment at transition.

	OVERALL n = 71	UC n = 44	CD n = 27
Therapy			
Mesalazine n (%)	47 (66.19)	32 (72.73)	15 (55.56)
Systemic Steroid n (%)	4 (5.63)	3 (6.81)	1 (3.70)
Topical acting oral steroids n (%)	2 (2.82)	2 (4.55)	0 (0.00)
Thiopurine n (%)	22 (40.84)	14 (31.82)	8 (29.63)
Methotrexate n (%)	0 (0.00)	0 (0.00)	0 (0.00)
Topic therapy n (%)	1 (1.41)	1 (2.27)	0 (0.00)
IFX n (%)	19 (26.76)	15 (34.09)	4 (14.81)
ADA n (%)	10 (14.08)	1 (2.27)	9 (33.33)
VDZ n (%)	0 (0.00)	0 (0.00)	0 (0.00)
USTE n (%)	1 (1.41)	0 (0.00)	1 (3.70)

IFX: Infliximab; ADA: Adalimumab; VDZ: Vedolizumab; USTE: Ustekinumab.

**Table 3:** Genetical results.

Analysis	Comparison Groups	Statistical Test	*p*-Value
NOD2/CARD15 variants: Pediatric vs. Adult CD Patients	Pediatric: 9 mut/11 wt Adult: 13 mut/97 wt	Chi-square Test	0.00048
Age at CD Onset: NOD2/CARD15 variants vs. Wild Type	Mean age ± SD Mut: 10.8 ± 2.08 WT: 13.2 ± 1.8	Student’s *t*-test	0.009492
Disease Phenotype (Paris Classification)	Mut: B2 = 5/B1 = 4 WT: B2 = 1/B1 = 10	Chi-square Test	0.024
Surgical Risk (NOD2/CARD15 variants vs. WT)	Mut: 4 Yes/5 No WT: 0 Yes/11 No	Fisher’s Exact Test	0.026

## Data Availability

The data supporting the conclusions of this article are provided within the article and are available from the corresponding authors upon request.

## Abbreviations

The following abbreviations are used in this manuscript:

MDPI	Multidisciplinary Digital Publishing Institute
DOAJ	Directory of open access journals
TLA	Three letter acronym
LD	Linear dichroism
